# Composition and Applications of *Aloe vera* Leaf Gel

**DOI:** 10.3390/molecules13081599

**Published:** 2008-08-08

**Authors:** Josias H. Hamman

**Affiliations:** Department of Pharmaceutical Sciences, Tshwane University of Technology, Private Bag X680, Pretoria, 0001, South Africa; E-mail: hammanjh@tut.ac.za; Tel.: +27 12 382 6397; Fax: + 27 12 382 6243

**Keywords:** *Aloe vera*, biological activities, absorption enhancement, skin permeation, excipient

## Abstract

Many of the health benefits associated with *Aloe vera* have been attributed to the polysaccharides contained in the gel of the leaves. These biological activities include promotion of wound healing, antifungal activity, hypoglycemic or antidiabetic effects anti-inflammatory, anticancer, immunomodulatory and gastroprotective properties. While the known biological activities of *A. vera* will be briefly discussed, it is the aim of this review to further highlight recently discovered effects and applications of the leaf gel. These effects include the potential of whole leaf or inner fillet gel liquid preparations of *A. vera* to enhance the intestinal absorption and bioavailability of co-administered compounds as well as enhancement of skin permeation. In addition, important pharmaceutical applications such as the use of the dried *A. vera* gel powder as an excipient in sustained release pharmaceutical dosage forms will be outlined.

## Introduction

Polysaccharides are found in abundance in Nature and are readily available from sources such as algae (e.g. alginates), plants (e.g. pectin, guar gum, mannan), microbes (e.g. dextran, xanthan gum) and animals (e.g. chitosan, chondroitin) and they can also be produced by means of recombinant DNA techniques. Monosaccharide polymers have many favourable properties such as high stability, non-toxicity, hydrophilicity, biodegradability, gel forming properties and ease of chemical modification [1,2]. An enormous variety in plant polysaccharide structural composition exists, which is not only associated with different plants, but also with the part of the plant that they originate from, such as the leaves, seeds, roots and tubers. The complexity and variety of polysaccharides can be explained by two unique structural features: firstly monosaccharides can be linked together in different ways (1→2, 1→3, 1→4, 1→5 and 1→6, in an *α-* or *β*-configuration) and secondly, due to the presence of branched side-chains [3].

Complex carbohydrates obtained from natural sources such as plants have shown diverse biological activities such as wound healing, enhancement of the reticuloendothelial system, stimulation of the immune system, treatment of tumours and effects on the hematopoietic system [4]. *Aloe vera* (L.) Burm.f. (*Aloe barbadensis* Miller) is a perennial succulent xerophyte, which develops water storage tissue in the leaves to survive in dry areas of low or erratic rainfall. The innermost part of the leaf is a clear, soft, moist and slippery tissue that consists of large thin-walled parenchyma cells in which water is held in the form of a viscous mucilage [5]. Therefore, the thick fleshy leaves of aloe plants contain not only cell wall carbohydrates such as cellulose and hemicellulose but also storage carbohydrates such as acetylated mannans [3].

*A. vera* has been used for many centuries for its curative and therapeutic properties and although over 75 active ingredients from the inner gel have been identified, therapeutic effects have not been correlated well with each individual component [6]. Many of the medicinal effects of aloe leaf extracts have been attributed to the polysaccharides found in the inner leaf parenchymatous tissue [7,8], but it is believed that these biological activities should be assigned to a synergistic action of the compounds contained therein rather than a single chemical substance [9].

*A. vera* is the most commercialised aloe species and processing of the leaf pulp has become a large worldwide industry. In the food industry, it has been used as a source of functional foods and as an ingredient in other food products, for the production of gel-containing health drinks and beverages. In the cosmetic and toiletry industry, it has been used as base material for the production of creams, lotions, soaps, shampoos, facial cleansers and other products.

In the pharmaceutical industry, it has been used for the manufacture of topical products such as ointments and gel preparations, as well as in the production of tablets and capsules [10,11]. Important pharmaceutical properties that have recently been discovered for both the *A. vera* gel and whole leaf extract include the ability to improve the bioavailability of co-administered vitamins in human subjects [12]. Due to its absorption enhancing effects, *A. vera* gel may be employed to effectively deliver poorly absorbable drugs through the oral route of drug administration. Furthermore, the dried powder obtained from *A. vera* gel was successfully used to manufacture directly compressible matrix type tablets. These matrix type tablets slowly released a model compound over an extended period of time and thereby showing potential to be used as an excipient in modified release dosage forms [13].

## *Aloe vera* leaf composition

### Structural composition

The aloe leaf can be divided into two major parts, namely the outer green rind, including the vascular bundles, and the inner colourless parenchyma containing the aloe gel. Description of the inner central part of the aloe leaf may sometimes be confusing, due to the different terms that are used interchangeably such as inner pulp, mucilage tissue, mucilaginous gel, mucilaginous jelly, inner gel and leaf parenchyma tissue. Technically, the term ‘pulp’ or ‘parenchyma tissue’ refers to the intact fleshy inner part of the leaf including the cell walls and organelles, while ‘gel’ or ‘mucilage’ refers to the viscous clear liquid within the parenchyma cells [7].

**Figure 1:**
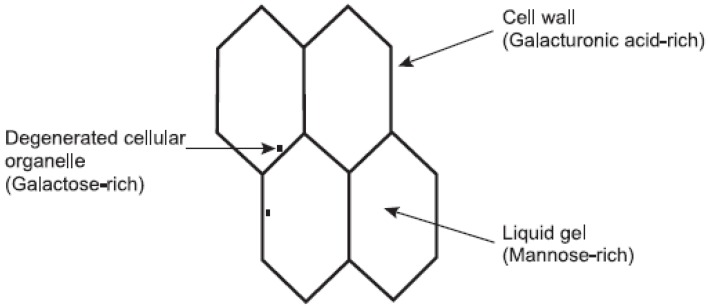
Schematic representation of *A. vera* leaf pulp structure and its components [8].

The three structural components of the *Aloe vera* pulp are the cell walls, the degenerated organelles and the viscous liquid contained within the cells. These three components of the inner leaf pulp have been shown to be distinctive from each other both in terms of morphology and sugar composition as shown in Figure 1 [8]. The raw pulp of *A. vera* contains approximately 98.5% water, while the mucilage or gel consists of about 99.5% water [10]. The remaining 0.5 – 1% solid material consists of a range of compounds including water-soluble and fat-soluble vitamins, minerals, enzymes, polysaccharides, phenolic compounds and organic acids [14]. It has been hypothesized that this heterogenous composition of the *Aloe vera* pulp may contribute to the diverse pharmacological and therapeutic activities which have been observed for aloe gel products [4].

### Chemical composition

Many compounds with diverse structures have been isolated from both the central parenchyma tissue of *A. vera* leaves and the exudate arising from the cells adjacent to the vascular bundles. The bitter yellow exudate contains 1,8 dihydroxyanthraquinone derivatives and their glycosides, which are mainly used for their cathartic effects [15]. The aloe parenchyma tissue or pulp has been shown to contain proteins, lipids, amino acids, vitamins, enzymes, inorganic compounds and small organic compounds in addition to the different carbohydrates. Some evidence of chemotaxonomic variation in the polysaccharide composition of aloes exists [7,16,17].

The large fluctuations in polysaccharide composition of *A. vera* fillet as found in the literature has been explained by the fact that the mannosyl residues are contained in a reserve polysaccharide with a significant seasonal influence, as well as large variations between cultivars in terms of the quantities of mannose-containing polysaccharides within the parenchyma cells [18]. The chemical constituents of *A. vera* leaves including the pulp and exudate are given in Table 1.

**Table 1:** Summary of the chemical composition of *A. vera* leaf pulp and exudate [7,9,17,18,19].

Class	Compounds
Anthraquinones/anthrones	Aloe-emodin, aloetic-acid, anthranol, aloin A and B (or collectively known as barbaloin), isobarbaloin, emodin, ester of cinnamic acid
Carbohydrates	Pure mannan, acetylated mannan, acetylated glucomannan, glucogalactomannan, galactan, galactogalacturan, arabinogalactan, galactoglucoarabinomannan, pectic substance, xylan, cellulose
Chromones	8-*C*-glucosyl-(2’-*O*-cinnamoyl)-7-*O*-methylaloediol A, 8-*C*-glucosyl-(*S*)-aloesol, 8-*C*-glucosyl-7-*O*-methyl-(*S*)-aloesol, 8-*C*-glucosyl-7-*O*-methyl-aloediol, 8-*C*-glucosyl-noreugenin, isoaloeresin D, isorabaichromone, neoaloesin A
Enzymes	Alkaline phosphatase, amylase, carboxypeptidase, catalase, cyclooxidase, cyclooxygenase, lipase, oxidase, phosphoenolpyruvate carboxylase, superoxide dismutase
Inorganic compounds	Calcium, chlorine, chromium, copper, iron, magnesium, manganese, potassium, phosphorous, sodium, zinc
Miscellaneous including organic compounds and lipids	Arachidonic acid, *γ*-linolenic acid, steroids (campestrol, cholesterol, *β*-sitosterol), triglicerides, triterpenoid, gibberillin, lignins, potassium sorbate, salicylic acid, uric acid
Non-essential and essential amino acids	Alanine, arginine, aspartic acid, glutamic acid, glycine, histidine, hydroxyproline, isoleucine, leucine, lysine, methionine, phenylalanine, proline, threonine, tyrosine, valine
Proteins	Lectins, lectin-like substance
Saccharides	Mannose, glucose, *L*-rhamnose, aldopentose
Vitamins	B1, B2, B6, C, *β*-carotene, choline, folic acid, *α*-tocopherol

### Polysaccharide composition

Polysaccharides make up most of the dry matter of the *A. vera* parenchyma. A storage polysaccharide, acetylated glucomannan, is located within the protoplast of the parenchyma cells and a variety of polysaccharides are present in the cell wall matrix. An overall carbohydrate analysis of the alcohol insoluble residues showed that the cell walls in the fillet of the aloe leaf hold mainly mannose-containing polysaccharides, cellulose and pectic polysaccharides whereas the skin of the leaf contains in addition significant quantities of xylose-containing polysaccharides [18,20]. 

Many investigators have identified partially acetylated mannan (or acemannan) as the primary polysaccharide of the gel, while others found pectic substance as the primary polysaccharide. As mentioned before, this discrepancy in polysaccharide composition was initially explained by differences in geographical locations of the plants and seasonal changes but later it was found that extraction and processing of the parenchyma tissue are also very important variables that contribute to the differences in the results. Other polysaccharides such as arabinan, arabinorhamnogalactan, galactan, galactogalacturan, glucogalactomannan, galactoglucoarabinomannan and glucuronic acid-containing polysaccharides have been isolated from the *Aloe vera* inner leaf gel part [8,19].

### Mannan

In general, mannans play a structural role in plants by acting as hemicelluloses that bind cellulose. They also fulfil a storage function as non-starch carbohydrate reserves in seeds and vegetative tissues. In addition, evidence was found that it may act as a signalling molecule in plant growth and development. Linear mannans are homopolysaccharides that are composed of linear chains of β-(1→4)-d-mannopyranosyl residues with less than 5% galactose [21].

Although different results on the composition of polysaccharides in aloe pulp have been described in the literature, the consensus among most authors is that acetylated glucomannan molecules are mainly responsible for the thick, mucilage like properties of the raw aloe gel. Acemannan found in *A. vera* gel is also known as carrysin and has a backbone of *β*-(1→4)-d-mannosyl residues acetylated at the C-2 and C-3 positions that exhibit a mannose monomer:acetyl ratio of approximately 1:1 and contains some side chains of mainly galactose attached to C-6. The molecular weights of these polysaccharides range from 30-40 kDa or greater and is usually as high as 1000 kDa in fresh aloe leaf material [18,21,22]. The repeating units of glucose and mannose exist in a ratio of 1:3, but other ratios of 1:6, 1:15 and 1:22 have also been reported. These discrepancies in glucose to mannose ratios have been explained by differences between species as well as due to sample processing and treatment [10,14].

**Figure 2:**
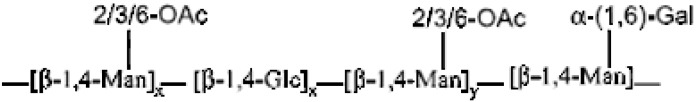
Chemical structure of acemannan [4].

In a study where the linkages between monomers in acemannan were analysed, the acemannan was treated with the enzyme endo-β-d-mannanase and the C-4 and C-6 resonances of the fractions were scrutinised using C^13^-NMR. This analysis demonstrated that acemannan contains a single-chain backbone of *β*-(1→4) mannose with *β*-(1→4) glucose inserted into the backbone and *α*-(1→6) galactose branching from the backbone, as illustrated in Figure 2 [4].

The *β*-(1→4)-glycosidic bond configuration of acemannan is an important consideration in terms of the therapeutic effects of *A. vera* gel, since humans lack the ability to enzymatically break down these bonds [14].The acemannan found in aloe is structurally unique that makes it a characteristic compound of aloe species amongst other well known plant mannans (which have distinct side-chains or are unacetylated and insoluble) [3].

Plant galactomannans are made up of *β*-(1→4)-d-mannopyranosyl residues containing side chains of single *α*-(1→6)-d-galactopyranosyl groups. True galactomannans are represented by those mannans that contain more than 5% by weight of d-galactose residues. The physiological function of plant galactomannans is to retain water by solvation, especially to prevent complete drying of seeds in regions with high temperatures. Glucomannans are polysaccharides that contain chains of randomly arranged *β*-(1→4)-d-manose and *β*-(1→4)-d-glucose residues in a ratio of 3:1. The backbone of galactoglucomannans consists of β-(1→4)-d-mannopyranosyl and *β*-(1→4)-d-glucopyranosyl residues with a *α*-(1→6)-d-galactopyranosyl and *O*-acetyl groups [21].

### Maloyl glucans

Three malic acid acylated carbohydrates were isolated from *A. vera* gel and characterised as 6-*O*-(1-l-maloyl)-*α-,β*-d-Glc*p* (termed veracylglucan A), *α*-d-Glcp-(1→4)-6-*O*-(1-l-maloyl)-*α-,β*-d-Glc*p* (termed veracylglucan B) and *α*-d-Glcp-(1→4)-tetra-[6-*O*-(1-l-maloyl)-α-d-Glcp-(1→4)]-6-*O*-(1-l-maloyl)-*α-,β*-d-Glc*p* (termed veracylglucan C).

**Figure 3:**
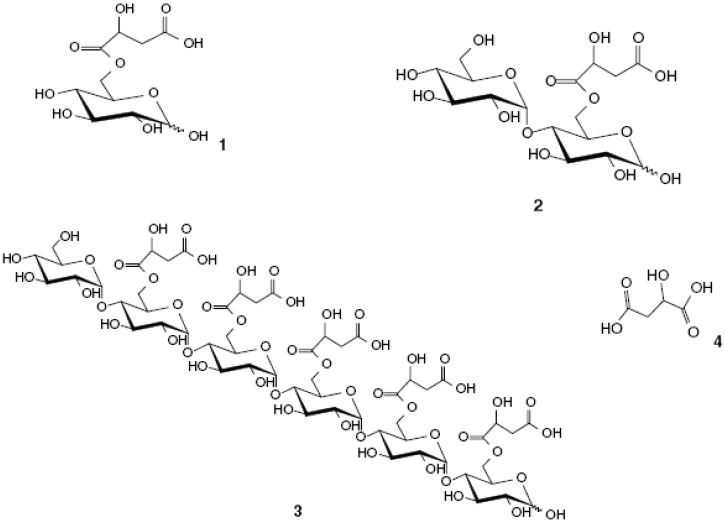
Chemical structures of veracylglucan A (1), veracylglucan B (2), veracylglucan C (3) and malic acid (4) [23].

Veracylglucan A (C_10_H_16_O_10_), with a molecular weight of 296 Da was only detected in very small quantities in the *A. vera* gel and was very unstable with hydrolysis of the ester group [6-*O*-(1-l-maloyl)-Glcp-] that occurred after only one week at a temperature of 7 °C. Veracylglucan B (C_16_H_26_O_15_) has a molecular weight of 458 Da and pH of 3.8, while veracylglucan C (C56H82O51) has a molecular weight of 1570 Da and a pH of 4.7 [23]. The chemical structure of the three different veracylglucans are shown in Figure 3.

### Pectic substance

Pectic substance is a term that refers to a group of closely related polysaccharides including pectin, pectic acid and arabinogalactan. Pectin is a polysaccharide consisting of *α*-(1→4) linked polygalacturonic acid with intra-chain rhamnose insertion, neutral sugar side-chains and methyl esterification [3].

### Arabinan and arabinogalactan

Arabinogalactan contains mainly arabinose and galactose, but also other sugars including glucuronic acid and/or galacturonic acid. Certain arabinans and arabinogalactans sometimes form the neutral side chains of pectins. Arabinogalactan is present in a much lower concentration in aloe gel compared to acemannan [3].

### Other polysaccharides

Aloeride is a polysaccharide that comprises only 0.015% of the crude *A. vera* juice material (dry weight). It has a molecular weight between 4 and 7 million Da with its glycosyl components containing glucose (37.2%), galactose (23.9%), mannose (19.5%) and arabinose (10.3%). Polyuronide has a molecular weight between 275 and 374 kDa, while that of aloeferon is 70 kDa. Another biologically active polysaccharide with a molecular weight between 420 and 520 kDa was isolated from aloe gel that comprises equal amounts of glucose and mannose [24].

## Effect of *Aloe vera* gel on biological membrane permeation

### Intestinal drug absorption enhancement

The effect of *A. vera* gel and whole leaf extract on the oral bioavailability of vitamins C and E was investigated in humans in a randomised, double-blind, cross-over clinical trial. Both the gel and whole leaf extract decreased the rate of vitamin C absorption, but the overall bioavailability (area-under-curve) of vitamin C was 3 times higher when administered with the aloe gel as compared to the control and the gel kept the level of this vitamin significantly higher (p ≤ 0.05) than the baseline even after 24 hours. The bioavailability of vitamin C administered in conjunction with the whole leaf extract was only 80 % compared to the control and the level returned to baseline after 24 hours. For vitamin E, the bioavailability was 3.7 times higher when administered with aloe gel and 2 times higher with the aloe whole leaf extract. The mechanism of action of the aloe products to improve the bioavailability of the vitamins was explained to be a possible protection effect against the degradation of the vitamins in the intestinal tract as well as binding of the polysaccharides to the vitamins and thereby slowing down the absorption rate [12].

**Figure 4:**
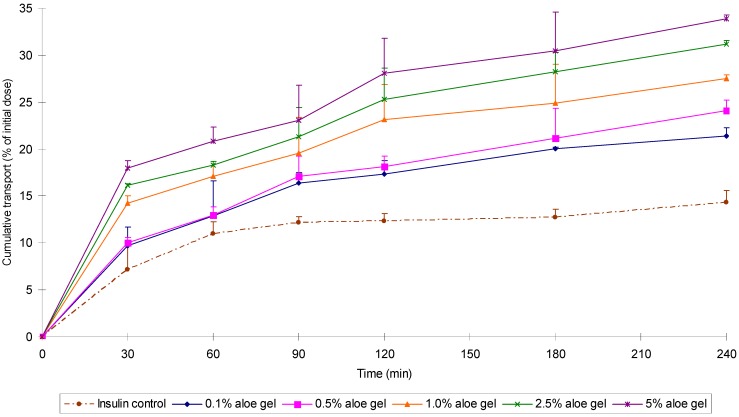
The effect of *A. vera* gel on the transport of insulin across Caco-2 cell monolayers at pH 7.4 [28].

It is well known that polysaccharides of natural origin such as chitosan are capable of enhancing the intestinal absorption of co-administered drugs by means of a transient opening of the tight junctions between adjacent epithelial cells to allow for paracellular transport across the intestinal epithelium [25,26]. In a recent *in vitro* study it was shown that both *A. vera* gel and whole leaf extract could decrease the transepithelial electrical resistance of intestinal epithelial cell monolayers (Caco-2), thereby indicating opening of the tight junctions between adjacent epithelial cells. The *A. vera* gel and whole leaf extract were also able to significantly increase the transport of the macromolecular peptide drug, insulin, across the Caco-2 cell monolayers. The cumulative transport of insulin in the absence (control) and presence of different concentrations of A. vera gel at pH 7.4 is depicted in Figure 4 [27,28].

Many potential therapeutic agents face the disadvantage of low bioavailability after oral administration due to poor membrane permeability [29]. Drug absorption enhancers are compounds capable of reversibly removing the resistance of the outer layers in the body with minimum tissue damage, thus allowing the drug to enter the blood circulation in sufficient quantities [30]. Although many compounds have been investigated for their drug absorption enhancing properties, some have been associated with cytotoxic effects and others were not efficient enough to ensure that therapeutic levels of poorly absorbable drugs are achieved [31]. Only limited information is currently available on the drug absorption enhancement activities of *A. vera* gel, but if it proves to be a safe and effective absorption enhancer *in vivo*, it could be used in novel dosage forms for the oral delivery of poorly absorbable drugs that are administered by means of injections.

### Skin penetration enhancement

Although there is a high interest in transdermal drug delivery, the poor penetration of drugs into the skin and low permeation across the skin severely hamper the use of this route of drug administration. Techniques for improving the transdermal delivery of drugs are based on the use of chemical penetration enhancers, novel vehicle systems and physical enhancement strategies such as iontophoresis, sonophoresis, ultrasound, microneedles, velocity based techniques and electroporation [32,33,34,35].

*A. vera* gel increased the *in vitro* skin penetration of compounds depending on their molecular weights, with an apparent inverse correlation between enhancement ratio and molecular weight of the compound. This penetration enhancement effect of the aloe gel was explained by a probable pull effect of complexes formed between the compound and the enhancing agent within the aloe gel, but it was stated that the proposed mechanism of action has to be further investigated and confirmed. Some constituents of the *A. vera* gel itself also penetrated the skin and this was interestingly dependent on the molecular weight of the co-applied compounds. The higher the molecular weight of the co-applied compound, the less of the gel components were transported across the skin. This was explained by the probable displacement of *A. vera* components from the penetration pathways and thereby it inhibits permeation of the gel components more effectively than the smaller compounds [36]. Similar to the discussion for intestinal drug absorption enhancement, *A. vera* gel could potentially be used as a penetration enhancement agent for the transdermal delivery of drugs if proven to be effective and safe.

### *Aloe vera* leaf gel as an excipient in modified release dosage forms

Gums and mucilages from natural origin that contain complex polysaccharides have found a wide range of pharmaceutical applications such as functional excipients in dosage forms, which include binders, disintegrants, emulsifiers, suspending agents, gelling agents and sustaining agents in modified release tablets. Furthermore, some natural gums and mucilages have been reported to modify the release of drugs from modified release dosage forms such as matrix type tablets [37].

Dried *A. vera* leaf gel (acetone precipitated component of the pulp) was directly compressed in different ratios with a model drug to form matrix type tablets, including ratios of 1:0.5; 1:1; 1:1.5; and 1:2. These matrix systems showed good swelling properties that increased with an increase of aloe gel concentration in the formulation. The directly compressed matrix type tablets also showed modified release behaviour with 35.45% and 30.70% of the dose released during the first hour and the remaining of the dose was released over a 6 hour period for those formulations containing the lower ratios of gel to drug, namely 1:0.5 and 1:1. The formulation that contained the highest ratio of gel to drug, namely 1:2 exhibited only a 23.25% drug release during the first hour with the remaining of the dose being released over an 8 hour period. The dried *A. vera* gel polysaccharide component therefore showed excellent potential to be used as an excipient in the formulation of direct compressible sustained-release matrix type tablets [13].

## Biological activities of *Aloe vera* leaf gel

It has been claimed that the polysaccharides in *A. vera* gel have therapeutic properties such as immunostimulation, anti-inflammatory effects, wound healing, promotion of radiation damage repair, anti-bacterial, anti-viral, anti-fungal, anti-diabetic and anti-neoplastic activities, stimulation of hematopoiesis and anti-oxidant effects [4,7,38].

On the other hand, there are a number of clinical reports that have found *A. vera* gel not effective in terms of the above mentioned therapeutic activities or even to cause undesirable effects such as retardation of wound healing. As mentioned before, these conflicting results could be due to the use of plants from different locations with variations in their chemical composition and also because of different isolation techniques that were used to extract compounds from the aloe leaf pulp. 

The importance of why the specific compounds that were isolated from a plant and then tested in a particular bioassay should be known can be demonstrated by the potential antagonistic and competitive activities between constituents. When the two maloyl glucans, namely veracylglucan B and C, were each individually evaluated for biological activities it was found that veracylglucan B demonstrated high anti-inflammatory and anti-proliferation effects, while veracylglucan C exhibited significant cell proliferative and anti-inflammatory activities. Therefore, if *A. vera* gel is tested in a wound healing experiment and it contains high amounts of veracylglucan B and is perhaps also contaminated with anthraquinones from the exudate, it will most probably result in retardation of wound healing. If the gel is obtained from a plant with higher concentrations of veracylglucan C, it would probably end in positive wound healing results [23].

Furthermore, the polysaccharides found in aloe gel are not stable, especially under stress conditions such as heat, the presence of acid and enzymatic activities. It has been suggested that a standardised method is necessary for production of aloe gel products to avoid degradation of the polysaccharides and thereby preventing the removal of high molecular weight molecules. This standardised and consistent production process is vital for preserving the natural biological activity of the aloe gel [39].

Some of the biological activities of *A. vera* gel will only be briefly described in this review as it has been comprehensively discussed elsewhere [19,22,38].

### Anti-diabetic effects

Several pre-clinical (in animals) and clinical (in humans) trials showed a blood glucose lowering effect for *A. vera* gel preparations in different forms (e.g. juice or as constituents in bread etc.), while other studies indicated that no change in glucose levels could be obtained. The differences in results of these *in vivo* studies can possibly be explained by differences in the way that the aloe mucilaginous gel was isolated and separated from the exudate anthraquinones. Furthermore, it is not always clear what constituent of the aloe leaf was tested in some studies, which makes it difficult to correlate the effect (or lack of effect) with the product tested [17,38].

In a study on streptozotocin-induced diabetic rats, oral administration of *A. vera* gel (alcohol insoluble residue extract) significantly reduced the fasting blood glucose, hepatic transaminases, plasma and tissue cholesterol, triglicerides, free fatty acids and phospholipids and in addition also significantly increased plasma insulin levels. The decreased plasma levels of high density lipoprotein cholesterol and increased levels of low density lipoprotein cholesterol in the streptozotocin-induced rats were restored to normal after treatment with gel extract [40].

From the findings of another study on streptozotocin-induced diabetic rats, it was suggested that the mechanism of action of *A. vera* extracts to reduce blood glucose levels is by enhancing glucose metabolism. It was further proposed that the glucose lowering effect could be explained by an anti-oxidant mechanism because it attenuated oxidative damage in the brains of streptozotocin-induced mice and reduced peroxidation levels in the kidneys of streptozotocin-induced diabetic rats [14].

### Immunomodulatory effects

A number of studies indicated immunomodulating activities of the polysaccharides in *A. vera* gel, and suggested that these effects occur via activation of macrophage cells to generate nitric oxide, secrete cytokines (e.g. tumour necrosis factor-alpha or TNF-α, interleukin-1 or IL-1, interleukin-6 or IL-6 and interferon-γ or INF-γ) and present cell surface markers [41,42,43]. Some immune reactions that seem to be specific for acemannan as compared to other polysaccharides include stimulation of the antigenic response of human lymphocytes as well as the formation of all types of leucocytes from both spleen and bone marrow in irradiated mice. However, some other immunomodulation effects were shown to be linked to glycoproteins, namely lectins, found in aloe gel [38].

In a study on the immunomodulatory properties of *A. vera*, it was shown that relatively high concentrations of acemannan are required to achieve modest activation of macrophages compared to crude *A. vera* juice, which suggested that there is another component in the juice responsible for the macrophage activation. Further investigation revealed that although it is present only in small amounts, its potency in terms of macrophage stimulation accounted fully for the activity obtained for the crude *A. vera* juice [24]. 

It was found that aloe gel can prevent suppression of local and systemic immunity to haptens and delayed type hypersensitivity responses to *Candida albicans* and alloantigen when applied after UV exposure. The mechanism of this immune protection effect by the polysaccharides in the gel differs from those described for anti-oxidants, anti-inflammatories and DNA-repair enzymes. Although anti-inflammatory agents have been identified in *A. vera*, the polysaccharides failed to reduce UV-induced edema and inflammation as well as to accelerate excision and repair of UV-induced cyclobutyl pyrimidine dimmers. In addition, anti-oxidants must be present in the skin before UV-irradiation to be effective while aloe polysaccharides are effective even when applied up to 24 h post UV exposure. The immune protection action therefore occurs at a step downstream from DNA damage and repair, possibly by modulating DNA-damage-activated signal transduction pathways. The mechanism of action of the polysaccharides was therefore explained by their effects on antigen presenting cells and the cytokine cascade [44].

### Anti-inflammatory effects

Inflammation is a reaction by the body due to injury and is characterised by swelling, pain, redness, heat and loss of function. This natural response can delay healing, but it may also be detrimental to suppress inflammation before its purpose is accomplished. The anti-inflammatory activity of mannose-6-phosphate is believed to resemble the effects observed for acetylated mannan in aloe gel. Aloe gel reduces inflammation that is induced by agents via promotion of prostaglandin synthesis as well as increased infiltration of leucocytes, but is less effective against inflammation caused by agents that produce allergic reactions [38].

The effects of aqueous, chloroform and ethanol extracts of *A. vera* gel were investigated on oedema in the rat paw as well as neutrophil migration into the peritoneal cavity induced by carrageenan. Both the aqueous and chloroform extracts were found to inhibit the oedema formation close to that of well established anti-inflammatory agents (i.e. indomethacin and dexamethasone). Furthermore, the anti-oedema effects of these two extracts correlated well with their abilities to decrease the number of neutrophils migrating into the peritoneal cavity. The ethanol extract did not show an effect on the oedema, but reduced the number of migrating neutrophils. Further experimentation on the mechanism of action suggested that the anti-inflammatory activity of the extracts of *A. vera* gel probably occurs via an inhibitory action on the arachidonic acid pathway through cyclooxygenase [15].

A study on *Helicobacter pylori*-infected rats showed that treatment with *A. vera* significantly reduced leukocyte adhesion and tumour necrosis factor α (TNF-α)levels. The results therefore suggest that *A. vera* show potential in the treatment of the inflammatory response of the gastric mucosa due to *H. pylori* infection [45].

### Anti-oxidant effects

It has been reported by several authors that different fractions of *A. vera* as well as unfractionated whole gel have anti-oxidant effects. Glutathione peroxidise activity, superoxide dismutase enzymes and a phenolic anti-oxidant were found to be present in *A. vera* gel, which may be responsible for these anti-oxidant effects. It was shown in two cell-free *in vitro* systems and by incubation with inflamed colorectal mucosal biopsies that *A. vera* gel has a dose-dependent anti-oxidant effect. The cell-free techniques used in this study assessed the scavenging of both superoxide and peroxyl radicals. The *A. vera* gel in a concentration of 1 in 50 also inhibited prostaglandin E_2_ production from inflamed colorectal biopsies, but had no effect on thromboxane B_2_ release [46].

### Wound healing effects

Wound healing is a response to injured tissue that results in the restoration of tissue integrity. It was shown that aloe gel could improve wound healing after topical and systemic administration in several studies, while others claimed no effect or even a delay in wound healing. Conflicting results may be explained by stability of the active ingredients as it was shown that the time of treatment after harvesting was an important factor that determined activity. Several mechanisms have been proposed for the wound healing effects of aloe gel, which include keeping the wound moist, increase epithelial cell migration, more rapid maturation of collagen and reduction in inflammation [38].

A 5.5 kDa glycoprotein that was isolated from *A. vera* showed an increase in cell migration and accelerated wound healing in a human keratinocyte monolayer. In a raft culture it exhibited stimulation of epidermal tissue formation as well as marked expression of proliferation markers on the immunohistochemical level. The enhanced wound healing effect and cell proliferation of this glycoprotein fraction was confirmed in hairless mice [47].

### Anti-cancer effects

The two fractions from aloes that are claimed to have anti-cancer effects include glycoproteins (lectins) and polysaccharides [38]. The anti-tumour activity of polysaccharides isolated from *A. vera* and specifically acemannan has been investigated in many *in vitro* models as well as in different animal species. Different studies indicated anti-tumour activity for *A. vera* gel in terms of reduced tumour burden, tumour shrinkage, tumour necrosis and prolonged survival rates. In addition to these effects, *A. vera* gel has also shown chemopreventative and anti-genotoxic effects on benzo[α]pyrene-DNA adducts [14]. One mechanism of action that was proposed for these anti-cancer effects of aloe polysaccharides is stimulation of the immune response [22].

### Effect on gastric acid secretion and ulcers

It has been claimed that *A. vera* gel has the ability to cure gastric ulcers or protect against its formation in both animals and humans. However, it was also shown that aloe gel could not prevent ethanol-induced gastric lesions in rats. The anti-ulcer activities of *A. vera* has been attributed to several possible mechanisms including its anti-inflammatory properties, healing effects, mucus stimulatory effects and regulation of gastric secretions [48].

The effect of ethanol-water extract of *A. vera* on gastric acid secretion and hydrochloric acid induced gastric mucosa damage was investigated in rats. The *A. vera* extract exhibited concentration dependent inhibition of gastric acid secretions, which was explained by direct interaction with the acid producing cells or possible interaction with H_2_-receptors on the parietal cells. Gastroprotective activity was only observed at the lowest dose tested. It was suggested that the *A. vera* extract possesses cytoprotection activity at this low concentration, therefore protection against mucosal injury by means of a mechanism different from gastric acid inhibition and neutralisation. Several hypotheses have been given for the mechanism of cytoprotection, namely increased mucus synthesis, increased mucosal blood flow and increased phospholipid content of the mucosal coating [49].

### Skin hydration effects

In a study where the moisturising effects of cosmetic formulations containing different concentrations of lyophilised *A. vera* gel were studied, showed that only formulations with higher concentrations (0.25 % w/w and 0.5 % w/w) increased the water content of the stratum corneum after a single application. When the formulations were applied twice daily for a period of 2 weeks, all the formulations (containing concentrations of 0.1 % w/w, 0.25 % w/w and 0.5 % w/w of *A. vera* gel powder) had the same effect. However, the transepidermal water loss was not changed by inclusion of the *A. vera* gel in the formulations compared to the vehicle used in the formulations. It was proposed that the *A. vera* gel containing products improved skin hydration possibly by means of a humectant mechanism [50].

### Hepatoprotective activities

An aqueous extract of dried aerial parts of *A. vera* significantly reduced hepatic damage induced by carbon tetrachloride in mice and reversed certain biochemical parameters. Histopathological studies confirmed the curative efficacy of the water extract of *A. vera* against carbon tetrachloride induced liver damage as indicated by reversal of centrilobular necrosis, macro-vascular fatty changes and scattered lymphomononuclear cell infiltrate in hepatic parenchyma. Furthermore, an increase in bile flow and bile solids as a result of treatment with the extract suggests stimulation of the secretary activity of the liver cells. The hepatoprotective action was also attributed to preserving the metabolising enzymes of the liver through an antioxidant activity [51].

### Antimicrobial activities

The activity of *A. vera* inner gel against both Gram-positive and Gram-negative bacteria has been demonstrated by several different methods [6]. Anthraquinones isolated from the exudate of *A. vera* have shown wide antimicrobial activity. The antibacterial activity of emodin against *Escherichia coli* was proposed to be mediated through inhibition of solute transport in membranes. Many anthraquinones have shown antiviral and/or virucidal effects on enveloped viruses [52].

## Conclusions

*A. vera* has a long history as a medicinal plant with diverse therapeutic applications. Although it was claimed that some of the biological activities of this plant can be attributed to the polysaccharides found in the leaf gel, it is a daunting task to link individual polysaccharides to specific therapeutic properties. Differences in plant composition due to geographic location as well as differences in gel extraction methods and sample preparation techniques have contributed to discrepancies in the results obtained from many studies in terms of the chemical composition and biological activities of *A. vera* leaf gel. Although some indications were found that a particular polysaccharide is effective when tested for a specific biological activity, it seems as if it is rather a combination of compounds that account for the health benefits of *A. vera* leaf gel. With technological developments in the field of analytical chemistry it has become easier to isolate and characterise the chemical components of the leaf gel and it is expected that more information in this regard will become available in the future at a faster rate. Interesting pharmaceutical applications such as intestinal absorption enhancement activities and skin penetration improvement effects have recently been shown for *A. vera* gel. The dried gel has also showed potential as an excipient in modified release matrix type tablets. More applications are discovered as research from different view points is conducted on this versatile plant to provide a better understanding of its composition and effects.
